# On the right TRK: from oncogene discovery to cancer therapeutics

**DOI:** 10.1093/annonc/mdz290

**Published:** 2019-11-18

**Authors:** M Barbacid

**Affiliations:** AXA-CNIO Professor of Molecular Oncology, Molecular Oncology Program, Centro Nacional de Investigaciones Oncológicas (CNIO) Spanish National Cancer Research Center, Madrid Spain

Circa 1983, transfection of mouse NIH3T3 cells with genomic DNA isolated from human tumours had already yielded the first three human cancer genes, all members of the RAS family. It was Friday evening and nobody else was in the lab. A fresh tumour biopsy obtained from a surgically removed colorectal carcinoma arrived at the lab of Stuart Aaronson. Since there was nobody to try to grow cells from this specimen, I decided to freeze it. Yet, before doing so, I could not resist the temptation to isolate DNA to ask whether fresh tumour samples might yield different cancer genes than cell lines or frozen tumour samples. Hence, I took a small piece, isolated its DNA and used it to transfect NIH3T3 cells shortly thereafter. It worked! But it was pure luck. Later, it became clear that “fresh” tumour samples contained the same oncogenes as those that were previously frozen.

This colorectal carcinoma contained an inversion within chromosome 1 that fused the amino-terminal domain of tropomyosin with the catalytic domain of a novel tyrosine protein kinase receptor. The resulting fusion oncogene was named TRK for tropomyosin receptor kinase, the first non-RAS oncogene isolated from a human tumour. Unfortunately, it never happened again, at least in our hands. Thus, we considered the TRK oncogene an oddity, a curiosity whose main contribution to science would be to serve as a probe to identify, a few years later, the signalling receptors for the NGF family of neurotrophins. These receptors, originally designated as TRKA, TRKB and TRKC, are now known as *NTRK1*, *NTRK2* and *NTRK3*, respectively.

However, the power of the new generation of sequencing techniques revealed that the TRK oncogene was not such an oddity after all. Although infrequent, TRK oncogenes formed by gene fusion events of each of the family members that result in their constitutive activation were identified in other common tumour types such as lung, breast and pancreatic carcinomas. In addition, they also occur with high frequency in some rare adult and paediatric tumours, such as secretory breast sarcomas and certain forms of thyroid cancer. These observations, despite expanding interest in the role of *NTRK* oncogenic fusions in human tumours, had limited impact in the cancer field. However, the generation of two potent inhibitors of TRK fusions, larotrectinib and entrectinib, has changed not only the clinical relevance of these oncogenes, but the way in which clinical oncologists must approach their patients.

Precision medicine has relied on the identification of selective mutations in tumours defined according to classical clinical parameters of tissue and cell type of origin followed by the identification of selective driver mutations. Lung cancer could be considered a classical paradigm of how clinicians carry out tumour stratification today. Once the tumour is classified according to histopathological parameters as a non-small-cell lung carcinoma, treatment selection is guided by the presence of known driver oncogenes such as mutant epidermal growth factor receptor or anaplastic lymphoma kinase (*ALK*) fusion for which there are selective inhibitors.

Availability of the TRK inhibitors larotrectinib* and entrectinib has made a change in the stratification methodology necessary by giving priority to the driver mutations over the tissue or cell of origin of the tumour. This new paradigm, known as the ‘tumor-agnostic’ strategy, was first used to identified tumours with microsatellite instability (MSI) for treatment with the anti-programmed cell death protein 1 monoclonal antibody, pembrolizumab, an immune checkpoint inhibitor. Without taking any value away from this pioneering therapeutic strategy, pembrolizumab does not identify any specific mutation. Its therapeutic activity on MSI tumours is likely to be a consequence of the higher number of ‘antigenic’ mutations present in these tumours. Indeed, pembrolizumab is used for the treatment of many other tumour types, such as lung and melanoma tumours, without MSI. In contrast, the use of larotrectinib and entrectinib invariably requires the identification of *NTRK* fusions: not a trivial task considering their low incidence in common malignancies. Yet, this diagnostic exercise can save lives. Clinical trials have revealed that more than two-thirds of patients carrying *NTRK* fusions treated with these inhibitors achieve high levels of tumour regression, regardless of tumour origin. In short, patients with certain tumour types, such as non-small-cell lung carcinomas or pancreatic adenocarcinomas, have a much higher chance of survival if they harbour *NTRK* gene fusions.

These observations underscore the need to carry out routine molecular identification of *NTRK* fusions using sequencing platforms. Identification of *EGFR* mutations or *ALK* fusions in lung adenocarcinomas only benefits 12% or 4% of patients, respectively. The incidence of *NTRK* fusions is a log lower. However, the therapeutic benefits provided by current TRK inhibitors certainly outweigh the increased expense of identifying *NTRK* fusions derived from their lower incidence. Development of potent and selective inhibitors against other oncogenic alterations is likely to lead to more instances of the use of tumour-agnostic strategies. Finally, for those of us who are still struggling to identify suitable therapies for *RAS* mutant tumours, the anecdote of isolation of the TRK oncogene provides comfort by reminding us that basic scientific discoveries will, sooner or later, serve to benefit patients with cancer. 

## Funding

This paper was published as part of a supplement financially supported by Bayer AG and Loxo Oncology, Inc., a wholly owned subsidiary of Eli Lilly and Company.

## Footnotes

* Note added in proof: The European Medicines Agency granted marketing authorisation for larotrectinib on 23 September 2019 as monotherapy for the treatment of adult and paediatric patients with solid tumours that display a neurotrophic tyrosine receptor kinase (*NTRK*) gene fusion, and who have disease that is locally advanced, metastatic or where surgical resection is likely to result in severe morbidity, and who have no satisfactory treatment options.

